# Abstracts reporting of HIV/AIDS randomized controlled trials in general medicine and infectious diseases journals: completeness to date and improvement in the quality since CONSORT extension for abstracts

**DOI:** 10.1186/s12874-016-0243-y

**Published:** 2016-10-13

**Authors:** Jean Joel R. Bigna, Jean Jacques N. Noubiap, Serra Lem Asangbeh, Lewis N. Um, Paule Sandra D. Sime, Elvis Temfack, Mathurin Cyrille Tejiokem

**Affiliations:** 1 Department of Epidemiology and Public Health, Centre Pasteur of Cameroon, Member of the International Network of the Pasteur Institute, Yaoundé, Cameroon; 2 Department of Medicine, Groote Schuur Hospital and University of Cape Town, Cape Town, South Africa; 3 Medical Diagnostic Center, Yaoundé, Cameroon; 4 Faculty of Medicine and Biomedical Sciences, University of Yaoundé 1, Yaoundé, Cameroon; 5 Internal Medicine Unit, Douala General Hospital, Douala, Cameroon; 6 Molecular Mycology Unit, Institut Pasteur of Paris, Paris, France

**Keywords:** Abstract, Randomized controlled trial, CONSORT, HIV, AIDS, Systematic review

## Abstract

**Background:**

Sufficiently detailed abstracts of randomized controlled trials (RCTs) are important, because readers often base their assessment of a trial solely on information in the abstract. We aimed at comparing reporting quality of RCTs in HIV/AIDS medicine before and after the publication of the 2008 CONSORT extension for abstracts and to investigate factors associated with better reporting quality.

**Methods:**

We searched PubMed/Medline for HIV/AIDS RCTs published between 2006–07 (Pre-CONSORT) and 2014–15 (Post-CONSORT) in 40 leading general medicine and infectious diseases journals. Two investigators extracted data and scored abstracts. The primary outcome was the adjusted mean number of items reported among the 17 required. Proportions of abstracts reporting each of 17 items were considered as secondary outcome. The adjustment was done for journal field, CONSORT endorsement, abstract format, type of intervention, journal impact factor and authorship. This study received no funding.

**Results:**

The adjusted mean number of reported items was 7.2 (95 % CI 6.6–7.7) in pre-CONSORT (*n* = 159) and 7.8 (95 % confidence interval [CI] 7.3–8.4) in post-CONSORT (*n* = 153) (mean difference 0.7; 95 % CI 0.1–1.2). Journal high impact factor (adjusted incidence rate ratio 2.16; 95 % CI 1.83–2.54), abstract with 13 authors or more (1.39; 95 % CI 1.07–1.79) and non-pharmacological intervention (1.19; 95 % CI 1.03–1.37) were independent factors for better reporting quality. There were significant improvements in reporting on participants, randomization, outcome results, registration and funding; regression for author contact; and no change for other items: title, design, interventions, objective, primary outcome, blinding, number randomized, recruitment, number analyzed, harms and conclusions.

**Conclusions:**

After the publication of the CONSORT extension for abstracts, the reporting quality of HIV/AIDS RCT abstracts in general medicine and infectious diseases journals has suboptimally improved. Thus, stricter adherence to the CONSORT for abstract are needed to improve the reporting quality of HIV/AIDS RCT abstracts.

**Electronic supplementary material:**

The online version of this article (doi:10.1186/s12874-016-0243-y) contains supplementary material, which is available to authorized users.

## Background

Randomized controlled trials (RCTs) which are designed to provide the best quality of evidence required for health care decisions [1, 2] should ideally be reported according to pre-defined standards. Most readers of articles reporting RCT usually start by making an initial assessment of the interest of the article after reading the content of the abstract, which subsequently guides the decision on whether to read the entire article or not [3]. With an overwhelming day-to-day workload, the continuous availability of large volumes of new scientific publications, limited access to many full-text articles (particularly in resource limited settings) [4], many health professionals tend to make recourse to information on abstracts to take health care decisions [3]. As such abstracts of trials reported in journals should contain sufficiently accurate and clear information that permits the reader to get a good synopsis of the findings of the trial [5]. Formerly, there was no standardized method of reporting RCT and this created many discordances in the reporting of RCTs. These discrepancies, stimulated the establishment of the Consolidated Standards of Reporting Trials (CONSORT) statement, the first of which was published in 1996 and revised in 2001 [6, 7], which aimed at “standardizing” and improving the way RCTs are reported [8]. In order to avoid inconsistencies between the content of the abstract and the full text of the article, as was seen in many RCT reports, an extension to the CONSORT statement was published in 2008, which provided a list of essential items to be included for reporting of abstracts of RCTs [9]. This statement comprises seventeen items distributed in eight sections which include: the title, authors contact details, trial design, methods (participants, interventions, objective, outcomes, randomization, blinding), results (numbers randomized, recruitment, numbers analyzed, outcome, harms), conclusions, trial registration and funding [9].

Following the publication of this CONSORT extension for abstract reporting, studies to assess the quality of abstract reporting in general medicine journals and other specific fields of medicine have been done. These studies have shown that there is substantial room for improvement in the adherence to these reporting guidelines [10–19].

However, as concerns RCTs in HIV/AIDS medicine, there are limited studies investigating the impact of CONSORT extension for abstract reporting checklist. In this field of HIV/AIDS where guidelines to tackle the pandemic are continually changing, adherence to this statement is particularly important, most especially as HIV/AIDS disproportionally affects resource limited settings (mainly sub-Saharan African countries) where most of the RCTs are done but most of health professionals do not have access to full-text articles required for health care decision making. We therefore, in the present study, using abstracts of the periods before and after the publication of CONSORT extension for abstracts, aim to first of all compare the overall reporting quality of RCTs in HIV/AIDS medicine published in general medicine and infectious diseases journals, and secondly determine the factors associated with better reporting quality.

## Methods

### Design

We conducted a systematic review of abstracts published in 2006, 2007, 2014 and 2015 in 20 leading general medicine and 20 leading infectious diseases journals based on 2014 impact factor.

### Data sources

We conducted a MEDLINE/PubMed search of all RCTs published in the years 2006–2007 and 2014–2015. The search strategy used MeSH terms like “randomized controlled trial” as publication type, journal names and HIV and AIDS as terms in title/abstract. We limited search for the following periods: (2006/01/01 to 2007/12/31 and 2014/01/01 to 2015/12/31). The choice of the time limit of 2014 and 2015 was arbitrary and based on the fact that 6 years after the publication of the first CONSORT statement for abstracts in 2008, is sufficient time for researchers to have become versed with the recommendations. Other types of articles (editorial, case reports, comment, observational study, review, meta-analysis and letters) were excluded. Searches were done regularly during the study period and the last one was conducted on February 26, 2016 as could be seen presented in Additional file 1.

### Studies selection

RCT abstracts where interventions were provided to HIV-infected patients were selected. These abstracts were included when participant allocation to interventions was described by the terms “random”, “randomly allocated”, “randomized”, “randomization” or another word in the abstract suggesting that participants were randomly distributed between the trial arms. We considered journals as CONSORT statement endorser if they referenced CONSORT statement in their ‘Instruction to authors/Submission guidelines’ or if they reference as endorser in the CONSORT webpage (http://www.consort-statement.org/about-consort/consort-endorsement/consort-endorsers---journals/). Two reviewers independently selected abstracts.

### Data extraction and covariates

Data extraction was independently done by two reviewers using a pre-made and pretested data extraction form in compliance with the items of the recommendations of the evaluation of RCTs using the CONSORT for abstracts [9]. Each question had a ‘yes’ or ‘no’ response for each item indicating whether the authors had reported it or not. Agreement between the two reviewers was measured using the Kappa statistic [20] and discrepancies were resolved by discussion among the authors or by arbitration of a third author. Other information collected from journals other than the CONSORT abstracts items, were journal name, type of abstract format (IMRAD [introduction, method, results and discussion] or eight-heading [objective, design, setting, participants, intervention, main outcome measure, results and conclusions] or one-block), type of interventions in the trial (pharmacological or non-pharmacological), number of authors, publication on behalf of a research collaboration group, and journal field (infectious diseases or general medicine).

### Main outcomes definition

The primary outcome was the number of items reported in each selected abstract. The secondary one was the proportion of abstracts reporting each of 17 recommended items.

### Statistical analysis

IBM Statistical Package for Social Science (IBM SPSS) version 23.0 for Windows (IBM Corp. Released 2014. IBM SPSS Statistics for Windows, Version 23.0. Armonk, NY: IBM Corp.) was used to code, enter and analyze data. Categorical variables were expressed as numbers with percentages (%). Continuous variables were expressed as means with standard deviation.

We expressed the number of items for each year as mean (standard deviation [SD]). We estimated mean differences using the independent two-sample *T*-test for unadjusted means and the generalized estimation equations (GEEs) for adjusted means [21]. All these mean differences were reported with 95 % confidence intervals (95 % CI). The *χ* ^2^ was used to compare compliance with the 17 items of CONSORT extension for abstracts between pre-CONSORT and post-CONSORT for abstracts publication. This was also done using GEE for adjusted analysis. We reported measures of association as odd ratios (OR) for univariate analyses and as adjusted odds ratios (aOR) for multivariate analyses both with their 95 % CI. A p-value < 0.05 was considered statistically significant.

Incidence rate ratios (IRRs) were computed to identify factors associated with better reporting in 2014–15 abstracts using GEE. For this analysis, Poisson distribution and unstructured correlation matrix were assumed. Univariate and multivariate analyses were conducted.

For GEE, adjustments were made for CONSORT endorser journal (yes/no), abstract format (IMRAD/8-heading/one-block), publication on behalf of a group (yes/no), number of authors (≤6/7–12/≥ 13), and journal field (general medicine/infectious diseases). Journal name has been used as grouping factor due to fact that they can similarity in articles published in the same journal. The adjustment was done for abstract format because there are previous studies reporting relationship between abstract format and quality reporting [15, 22]. The adjustment was also done for number of authors and publication on behalf a group because it was reported an association with higher quality of work with number of collaborators [23, 24].

## Results

### General characteristics of selected abstracts

**Fig. 1:**
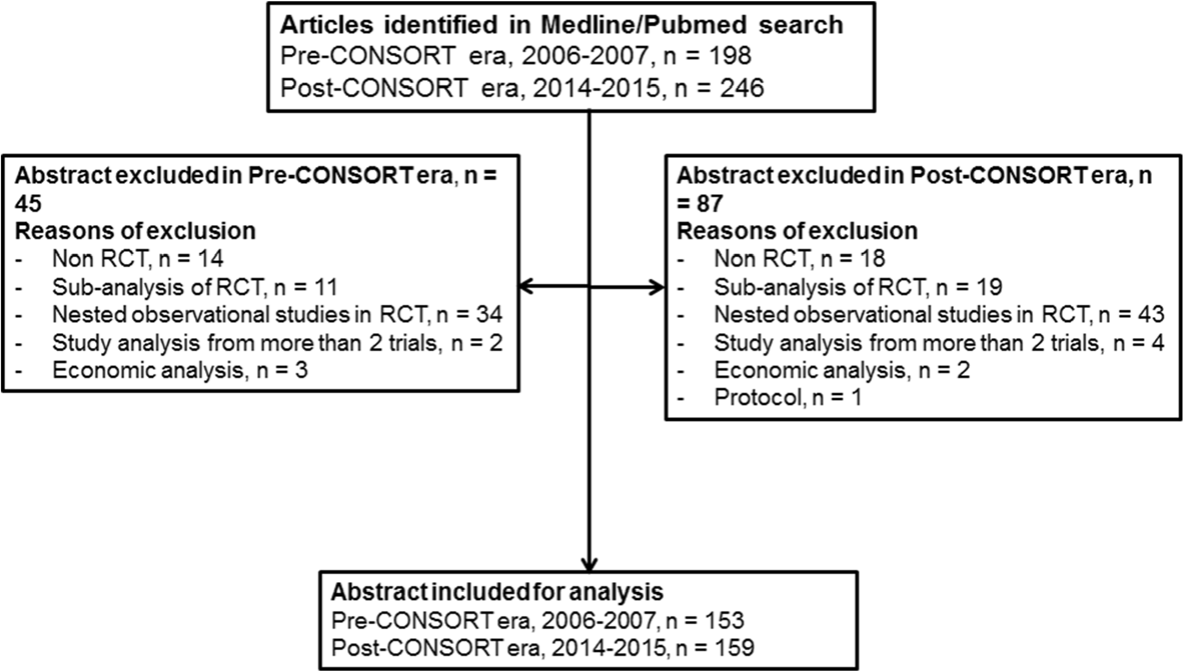
Flow chart of included studies

Our search yielded 444 articles of which 95 were published in 2006, 103 in 2007, 140 in 2014 and 106 in 2015. One hundred and thirty-two abstracts did not meet our inclusion criteria, hence were excluded. We therefore included 76 abstracts from 2006, 83 from 2007 (2006–07, *n* = 159), 81 from 2014 and 72 from 2015 (*n* = 153) as shown in the flow diagram (Fig. 1).

The inter-observer agreement value was 0.86 for abstract selection, 0.71 for overall rating of abstract reporting quality, and varied between 0.74 and 0.98 for each items of CONSORT for abstracts.

All articles came from 19 of 40 journals included in search strategy. Interventions were pharmacological/vaccine in 76 % (238/312) of the abstracts. The IMRAD format was used in 85 % (266/312) of abstracts. Among all the journals, 84 % of abstracts (262/312) were from journals which endorsed CONSORT, 80 % (248/312) were from infectious diseases journals, 63.5 % (198/312) were published on behalf of a group, and 75 % (233/312) were published in journals with impact factor less than 10. The mean number of authors among articles was 12.2 (SD 6.8) (Table 1).

**Table 1:** Distribution of HIV abstracts by year and characteristics

	2006–2007 *n* = 159	2014–2015 *n* = 153	All *N* = 312
Journals			
- AIDS	50 (31.4)	20 (13.1)	70 (22.4)
- AIDS Patient Care STDS	2 (1.3)	7 (4.6)	9 (2.9)
- Annals of Internal Medicine	2 (1.3)	2 (1.3)	4 (1.3)
- Archives of Internal Medicine	4 (2.5)	0	4 (1.3)
- BMC Medicine	0	3 (2.0)	3 (1.0)
- British Medical Journal	3 (1.9)	2 (1.3)	5 (1.6)
- Clinical Infectious Diseases	12 (7.5)	8 (5.2)	20 (6.4)
- HIV Medicine	8 (5.0)	4 (2.6)	12 (3.8)
- International Journal of Antimicrobial Agents	0	1 (0.7)	1 (0.3)
- JAMA Internal Medicine	0	1 (0.3)	1 (0.3)
- Journal of Acquire Immune Deficiency Syndrome	39 (24.5)	40 (26.1)	79 (25.3)
- Journal of Antimicrobial Chemotherapy	1 (0.6)	3 (2.0)	4 (1.3)
- Journal of Infectious Diseases	14 (8.8)	10 (6.5)	24 (7.7)
- Journal of the American Medical Association, The	2 (1.3)	6 (3.9)	9 (2.9)
- Journal of the International AIDS Society	0	3 (2.0)	3 (1.0)
- Lancet Infectious Diseases, The	0	20 (13.1)	20 (6.4)
- Lancet, The	14 (8.8)	7 (4.6)	21 (6.7)
- New England Journal of Medicine, The	6 (3.8)	7 (4.6)	13 (4.2)
- Pediatric Infectious Diseases Journal, The	0	6 (3.9)	6 (1.9)
- PLoS Medicine	2 (1.3)	3 (2.0)	5 (1.6)
Mean number of authors	11.1 (4.6)	13.3 (8.4)	12.2 (6.8)
- 0–6	19 (12.0)	20 (13.1)	39 (12.5)
- 7–12	97 (61.0)	67 (43.8)	164 (52.6)
- 13 and more	43 (27.0)	66 (43.1)	109 (34.9)
Journal impact factor			
- <10	126 (79.2)	107 (69.9)	233 (74.7)
- ≥10	33 (20.8)	46 (30.1)	79 (25.3)
Pharmacological/Vaccine intervention			
- Yes	129 (81.1)	109 (71.2)	238 (76.3)
- No	30 (18.9)	44 (28.8)	74 (23.7)
Abstract format			
- IMRAD	137 (86.2)	129 (84.3)	266 (85.3)
- Eight-heading	11 (6.9)	14 (9.2)	25 (8.0)
- One-block	11 (6.9)	10 (6.5)	21 (6.7)
CONSORT Endorsers journals			
- Yes	137 (86.2)	125 (81.7)	262 (84.0)
- No	22 (13.8)	28 (18.3)	50 (16.0)
Journal field			
- Infectious diseases	126 (79.2)	122 (79.7)	248 (79.5)
- General medicine	33 (20.8)	31 (20.3)	64 (20.5)
Publication on behalf of a group			
- Yes	97 (61.0)	52 (34.0)	114 (36.5)
- No	62 (39.0)	101 (66.0)	198 (63.5)

There were no abstract for the following journals included in search strategy: AIDS Review, The American Journal of Medicine, American Journal of Preventive Medicine, Annals of Family Medicine, Annals of Medicine, Canadian Medical Association Journal, Clinical Microbiology and Infection, Cochrane Database of Systematic Reviews, Current Opinion in HIV and AIDS, Current Opinion in Infectious Diseases, Emerging Infectious Diseases journal, Eurosurveillance, Infection and Immunity, Infection Control and Hospital Epidemiology, Journal of Cachexia Sarcopenia Muscle, Journal of infection, Journal of Internal Medicine, Mayo Clinic Proceedings, Medicine and Translational Research

### Overall quality of reporting

Only 1 % (2/153) of abstracts reported all items in the 2014–15 period. The mean number of items reported in 2006–07 (6.2; SD = 2.5; Median 6; Range 1–14) differed significantly from that reported in 2014–15 (7.7; SD = 4.0; Median 7; Range 1–17), mean difference (MD): 1.6; 95 % CI 0.8–2.3; p <0.001.

In the GEE analysis, after adjusting for covariates among the type of intervention, journal endorser of CONSORT, format of abstract, journal field, numbers of authors, journal impact factor and publication on a behalf of a group, the adjusted mean number of items reported in 2006–07 (7.2; 95 % CI 6.6–7.7) differed significantly from that reported in 2014–15 (7.8; 95 % CI 7.3–8.4), adjusted MD: 0.7; 95 % CI 0.1–1.2; p = 0.015.

### Quality of reporting of each item

In univariate analysis, there was improvement from 2006–07 to 2014–15 for ‘title’, ‘trial design’, ‘participants’, ‘objective’, ‘randomization’, ‘blinding’, ‘recruitment’, ‘number analyzed’, ‘outcome’, ‘conclusion’, ‘trial registration’ and ‘funding’. However, the quality of abstracts decreased for ‘author contact’. After adjustment, the quality of abstract remained improved for the following items: ‘participants’ (aOR 1.92; 95 % CI 1.05–3.51), ‘randomization’ (p < 0.001), ‘outcome’ of results section (aOR 2.26; 95 % CI 1.17–4.35), ‘trial registration’ (aOR 8.32; 95 % CI 3.66–10.88) and ‘funding’ (p < 0.001). From the 2006–07 period to the 2014–15 period, the quality of the item ‘author contact’ decreased (aOR 0.06; 95 % CI 0.03–0.12) (Table 2).

**Table 2:** Crude and adjusted odds ratios for adherence to the 12 items of the CONSORT statement for HIV/AIDS abstracts in 2014–2015 compared to 2006–2007

Item	Criteria	Items reported, *n* (%)		Data collection period (2014–15 versus 2006–07)			
		2014–15	2006–07	Univariate analysis^a^		Multivariate analysis^b^	
		*n* = 153	*n* = 159	Odds ratio (95 % CI)	*P*	Adjusted odds ratio (95 % CI)	*P*
Title	Identification of the study as randomized	103 (67.3)	80 (50.3)	2.03 (1.30–3.22)	.002	1.57 (0.93–2.65)	.090
Author contact	Contact details for the corresponding author including both postal and email addresses	28 (18.3)	110 (69.2)	0.10 (0.06–0.17)	< .001	0.06 (0.03–0.12)	< .001
Trial design	Description of the trial design (eg, parallel, cluster, non-inferiority, parallel, N-of-1 trial, etc.)	45 (29.4)	29 (18.2)	1.87 (1.10–3.18)	.020	1.56 (0.86–2.83)	.142
Methods							
- Participants	Eligibility criteria for participants and the settings where the data were collected	73 (47.7)	45 (28.3)	2.31 (1.45–3.69)	< .001	1.92 (1.05–3.51)	.034
- Interventions	Interventions intended for each group	142 (92.8)	140 (88.1)	1.75 (0.80–3.82)	.154	2.26 (0.99–5.12)	.052
- Objective	Specific objective or hypothesis	126 (82.4)	115 (72.3)	1.79 (1.04–3.07)	.035	1.31 (0.72–2.39)	.384
- Outcome	Clearly defined primary outcome	70 (45.8)	74 (46.5)	0.97 (0.62–1.51)	.889	0.69 (0.39–1.22)	.200
- Randomization	How participants were allocated to interventions	19 (12.4)	0	Not estimable	< .001	Not estimable	< .001
- Blinding (masking)	Whether or not participants, care givers and those assessing the outcomes were blinded to group assignment	18 (11.8)	3 (1.9)	6.93 (2.00–24.05)	< .001	4.10 (0.81–20.46)	.087
Results							
- Number randomized	Number of participants randomized to each group	64 (41.8)	56 (35.2)	1.32 (0.84–2.09)	.230	1.15 (0.69–1.91)	.601
- Recruitment	Trial status	50 (32.7)	31 (19.5)	2.00 (1.19–3.36)	.008	1.48 (0.82–2.68)	.192
- Number analyzed	Number of participants analyzed in each group	65 (42.5)	40 (25.2)	2.20 (1.36–3.55)	.001	1.69 (0.94–3.04)	.077
- Outcome	For the primary outcome, a result for each group and the estimated effect size and its precision	65 (42.5)	40 (25.2)	2.20 (1.36–3.55)	.001	2.26 (1.17–4.35)	.015
- Harms	Important adverse events or side-effects	78 (51.0)	75 (47.2)	1.16 (0.75–1.82)	.501	1.15 (0.65–2.02)	.632
Conclusions	General interpretation of the results	118 (77.1)	102 (64.2)	1.88 (1.15–3.10)	.012	1.59 (0.92–2.74)	.094
Trial registration	Registration number and name of trial register	84 (54.9)	38 (23.9)	3.88 (2.39–6.29)	< .001	8.32 (3.66–10.88)	< .001
Funding	Source of funding	34 (22.2)	1 (0.6)	45.14 (6.09–334.48)	< .001	Not estimable	< .001

*CI* confidence interval

^a^Chi squared tests

^b^Generalized estimation equations with journal as grouping variable: adjustment has been made for journal impact factor (<10 versus ≥ 10), journal field (general medicine versus infectious diseases), CONSORT endorser journal (yes versus no), abstract format (IMRAD/eight-heading/one-block), type of intervention (pharmacological versus non pharmacological), number of authors (less than 6/7–12/more than 13); expect for title and author contact in which abstract format was not considered

### Factors associated with better reporting quality in 2014–2015 abstracts

In univariate analysis, factors associated with better reporting included publishing in general medicine journal, publishing in CONSORT endorser journals, structured abstract (IMRAD or 8-heading), high impact journal, more than 13 authors and publishing on behalf of a research collaboration group. In multivariate analysis, factors independently associated with better reporting of abstracts in 2014–15 included publishing on non-pharmacological/vaccine intervention (aIRR 1.19; 95 % CI 1.03–1.37), more than 13 authors (aIRR 1.39; 95 % CI 1.07–1.79) and high journal impact factor (aIRR 2.16; 95 % CI 1.83–2.54) (Table 3).

**Table 3:** Unadjusted and adjusted incidence rate ratios for the total number of CONSORT extension for HIV abstract items reported

Variables	Total number of items reported			
	Univariate analysis		Multivariate analysis	
	Incidence rate ratio (95 % CI)	*p*	Adjusted incidence rate ratio (95 % CI)	*p*
Journal field				
- General medicine	33.11 (9.72–112.73)	< .001	1.19 (1.00–1.41)	.052
- Infectious diseases			1	
CONSORT endorsement				
- Non endorser journals			1	
- Endorser journals	11.22 (3.90–32.27)	< .001	1.01 (0.85–1.21)	.900
Abstract format				
- One-block			1	
- IMRAD	20.69 (6.94–61.61)	< .001	1.11 (0.81–1.51)	.524
- Eight-heading	57.81 (14.64–228.23)	< .001	1.28 (1.00–1.67)	.061
Pharmacological/Vaccine intervention				
- Yes			1	
- No	0.40 (0.11–1.42)	.155	1.19 (1.03–1.37)	.015
Journal impact factor				
- <10			1	
- ≥10	511.06 (186.40–1401.21)	< .001	2.16 (1.83–2.54)	< .001
Number of authors				
- Less than 6			1	
- 7–12	3.81 (0.75–19.41)	.108	1.18 (0.98–1.48)	.140
- More than 13	89.74 (15.30–526.36)	< .001	1.39 (1.07–1.79)	.013
Publication on behalf of a collaboration research group				
- No			1	
- Yes	13.09 (3.50–48.97)	< .001	0.99 (0.88–1.11)	.867

*CI* confidence interval, *CONSORT* consolidated standards of reporting trials, *IMRAD* introduction, methods, results and discussion

## Discussion

This study aimed to assess, according to the CONSORT for abstract checklist, the differences in the reporting quality of abstracts of RCTs in HIV/AIDS medicine published in general medicine and infectious diseases journals before (in the years 2006–07) and after (in the years 2014–15) the publication of the CONSORT extension for abstracts [9]. Our findings demonstrate that for some items (participants, randomization, outcome of results section, trial registration and funding), the reporting quality of HIV/AIDS RCT abstracts has improved significantly in the post-CONSORT era as compared to the pre-CONSORT era, while for others the quality has remained unchanged or regressed (authors contact). These results are consistent with previous studies that have reported inconsistencies and patterns of non-compliance to the CONSORT for abstracts guidelines in most of journals [10–19].

The mean number of items reported increased significantly from 2006–07 to 2014–15. This is in line with the average of 3 more items reported in RCT abstracts in top-tiers of general medicine journals since 2008 as found in a recent study [19]. High impact factor was independently associated with more adherence to CONSORT abstract items as in a study in which high impact factor is associated with high quality reporting for CONSORT items [15, 25–30]. Many people and institutions consider journal impact factor as the reflect of articles’ quality [31]. High number of authors was also independently associated with more adherence to CONSORT abstract items as in previous studies [23, 24]. One can argue that the involvement of a large number of authors in an article could improve quality. Many authors would increase the chances that one or more of the authors ensure to the respect of the reporting guidelines. We also found non pharmacological/vaccine intervention in trial a factor for better reporting. This finding requires more investigation to understand this relationship.

As a whole, the reporting of some items improved significantly over time: more abstracts in 2014–15 as compared to 2006–07 identified the study as randomized in the title, provided details on the trial design, randomization, blinding, trial registration and funding. For most abstract elements there was progress in reporting even though this was not significant. One of the main reasons that can explain the improvement of the overall reporting quality of abstracts found in our study is the endorsement of the CONSORT statement by most of the journals surveyed. Indeed, 84 % of articles included in our analysis were published in journals that endorsed CONSORT. A systematic review that examined the effectiveness of the CONSORT statement in journals that have formally endorsed it concluded that journals’ adoption of CONSORT is associated with improved reporting of RCTs [8]. Moreover, another study has also shown that the endorsement of recommendation and effective implementation of CONSORT guidelines lead to the improvement of reporting quality of abstracts [12]. In line with these studies, our findings stress the need to expand journal endorsement and implementation of the CONSORT extension for abstracts before manuscript submission (in journal submission guidelines), during review process and editorial assessment. Authors should be required to submit a completed CONSORT checklist with each manuscript with an emphasis for CONSORT extension for abstracts [32]; reviewers should assess the completeness of abstract reporting; and final manuscript copyediting would surely improve abstract reporting quality [19].

Despite this overall improvement, the quality of reporting of some items remained sub-optimal, consistent with findings from other studies [10–19]. Poorly reported items specifically include methods of randomization and allocation concealment, and blinding. These items are essential methodological elements which are critical, therefore adequate standardized reporting is indispensable so as to demonstrate that selection bias was avoided, thereby allowing unequivocal interpretations of the study findings [5]. Nevertheless, less abstracts in 2014–15 reported corresponding authors’ details (postal and email addresses). This is a major drawback when readers need to contact authors for further information on the study. This depends on journal indexing policy, rather than a deficiency in reporting. In addition, author contact details are more relevant for conference abstracts, none of which were included in this study [5].

Though we found that there has been some improvement in HIV RCT abstract reporting, our study had some limitations. We not investigated others factors that can influence reporting quality. These non-investigated factors include abstract word count, types of utilization of CONSORT guidelines in the ‘instructions for authors’ section of journals, and awareness of the CONSORT statement. Furthermore, this study reports on the adequacy of reporting using the CONSORT checklist items, but the accuracy of reporting cannot be assured because a comparison of the abstract and the full text was beyond our scope.

## Conclusions

The reporting quality of RCT abstracts in HIV/AIDS medicine in general medicine and infectious diseases journals has sub-optimally improved after the publication of the CONSORT extension for abstracts. This suboptimal improvement is associated with journal high impact factor, high number of authors and non-pharmacological/vaccine intervention in the trial. However, there is still much to do for improvement to meet the standards of the CONSORT for abstracts guidelines. Journal endorsement and more strict adherence to the CONSORT for abstracts standards by both authors and journal editors will contribute to better RCT reports in HIV/AIDS medicine. Further researches are necessary to investigate why authors, reviewers, journal editors, funders, institutions and readers not fully adhere to CONSORT guidelines.

## Additional file

Additional file 1: Search strategy for HIV RCTs published in 2006–2007 and 2014–2015 in leading general medicine and infectious diseases journals. (DOCX 14 kb)

## Abbreviations

(a)OR: (adjusted) odds ratio
AIDS: Acquired immunodeficiency syndrome
CI: Confidence interval
CONSORT: Consolidated standards of reporting trials
GEE: Generalized estimation equations
HIV: Human immunodeficiency virus
IMRAD: Introduction, method, results and discussion
IRR: Incidence rate ratio
RCT: Randomized controlled trials
SD: Standard deviation
