# Supplementary material for: Abstracts reporting of HIV/AIDS randomized controlled trials in general medicine and infectious diseases journals: completeness to date and improvement in the quality since CONSORT extension for abstracts
Source: BMC Med Res Methodol. 2016 Oct 13;16:138. doi: 10.1186/s12874-016-0243-y (PMC5064790; doi:10.1186/s12874-016-0243-y)
Supplement: Additional file 1: — Search strategy for HIV RCTs published in 2006–2007 and 2014–2015 in leading general medicine and infectious diseases journals. (DOCX 14 kb) [file 12874_2016_243_MOESM1_ESM.docx]

**Additional file 1. Search strategy for HIV RCTs published in 2006-2007 and 2014-2015 in leading general medicine and infectious diseases journals**

| **Search** | **Search terms** |
| --- | --- |
| #1 | (Randomized controlled trial[pt]) |
| #2 | (Editorial[pt]) OR (Case reports[pt]) OR (Comment[pt]) OR (Observational study[pt]) OR (Review[pt]) OR (Meta-analysis[pt]) OR (Letter[pt]) OR (Interview[pt]) OR (Lectures[pt]) OR (Retraction of publication[pt]) OR (retracted publication[pt]) |
| #3 | #1 NOT #2 |
| #4 | (“New England Journal of Medicine”[journal]) OR (“Lancet”[journal]) OR (“JAMA”[journal]) OR (“Ann Intern Med”[journal]) OR (“BMJ”[journal]) OR (“Arch Intern Med”[journal]) OR (“PLOS Med”[journal]) OR (“JAMA intern med”[journal]) OR (“J Cachexia Sarcopenia Muscle”[journal]) OR (“BMC Med”[journal]) OR (“Mayo Clin Proc”[journal]) OR (“J Intern Med”[journal]) OR (“Cochrane Database Syst Rev”[journal]) OR (“CMAJ”[journal]) OR (“Medicine”[journal]) OR (“Am J Med”[journal]) OR “(Ann Med”[journal]) OR (“Ann Fam Med”[journal]) OR (“Am J Prev Med”[journal]) OR (“Transl Res”[journal]) |
| #5 | (“Lancet Infect Dis”[journal]) OR (“Clin Infect Dis”[Journal]) OR (“Emerg Infect Dis”[journal]) OR (“J Infect Dis”[journal]) OR (“Clin Microbiol Infect”[journal]) OR (“Eurosurveillance”[journal]) OR (“AIDS”[journal] OR (“J Antimicrob Chemother”[journal]) OR (“J int AIDS Soc”[journal]) OR (“Curr Opin Infect Dis”[journal]) OR (“Curr Opin HIV AIDS”[journal]) OR ("JAIDS”[journal]) OR (“J infection”[journal]) OR (“Int J Antimicrob agents”[journal]) OR (“AIDS Rev”[journal]) OR (“Infect Immun”[journal]) OR (“Infect Control Hosp Epidemiol”[journal]) OR (“Pediatr Infect Dis J”[journal]) OR (“HIV Med”[journal]) OR (“AIDS Patient Care STDS”[journal]) |
| #6 | #4 OR #5 |
| #7 | (HIV[tiab]) OR (AIDS[tiab]) |
| #8 | ("2006/01/01"[Date - Publication] : "2007/12/31"[Date - Publication]) OR ("2014/01/01"[Date - Publication] : "2015/12/31"[Date - Publication]) |
| #9 | #3 AND #6 AND #7 AND #8 |
